# Effect of fermentation on the constituents in the branches and leaves of *Taxus media* and non-small cell lung cancer

**DOI:** 10.3389/fphar.2024.1449498

**Published:** 2024-10-23

**Authors:** Xing Guo, Rui-Sheng Wang, Zhen-Ling Zhang, Hong-Wei Zhang, Sheng-Chao Wang, Shuai Zhang, Ya-Ning Wu, Ya-Jing Li, Jun Yuan

**Affiliations:** ^1^ School of Pharmacy, Henan University of Chinese Medicine, Zhengzhou, China; ^2^ Collaborative Innovation Center of Research and Development on the Whole Industry Chain of Yu-Yao, Henan Province, Zhengzhou, China; ^3^ Henan Engineering Technology Research Center for Integrated Traditional Chinese Medicine Production, Zhengzhou, China; ^4^ Henan Engineering Research Center of Traditional Chinese Medicine Characteristic Processing Technology, Zhengzhou, China

**Keywords:** non-small cell lung cancer1, Taxus media2, fermentation3, antitumor activity4, herbal medicine5

## Abstract

**Introduction:**

Non-small cell lung cancer (NSCLC) is a prominent lung cancer disease worldwide. Currently, commonly used methods, such as surgery and radiotherapy, have significant side effects. Traditional Chinese medicine (TCM) has become a research hotspot because of its safe and effective characteristics. The branches and leaves of *Taxus media* are abundant in antitumor active compounds, and there has been no research conducted as yet regarding its anti–lung cancer molecular mechanism.

**Objective:**

The aim of this study is to investigate the antitumor activity of two samples before and after fermentation of *T. media*, and to research the molecular mechanism of its inhibitory effect on NSCLC.

**Methods:**

The chemical composition of pre-fermentation *T. media* (TM) and post-fermentation *T. media* qu (TMQ) were investigated using UHPLC-Q-Qrbitrap HRMS and high-performance liquid chromatography (HPLC). The anti-lung cancer activities of TM and TMQ were compared using an A549-induced tumor mouse model. An enzyme-linked immunosorbent assay (ELISA), hematoxylin and eosin (H&E) staining, immunohistochemistry, and immunofluorescence were used to determine the of TMQ mechanism of action.

**Results:**

The results indicated that TM and TMQ contained 83 compounds, consisting primarily of flavonoids, organic acids, and taxanes. Both taxanes and flavonoids in TMQ were higher than that in TM. Both TM and TMQ effectively inhibited the tumor growth in non-small cell lung cancer (NSCLC), and the inhibition rate was greater in TMQ (57.24%) than in TM (49.62%). TMQ administration downregulated the tumor necrosis factor-α (TNF-α), interleukin-6 (IL-6), and the glutathione (GSH) level and upregulated interferon-γ (IFN-γ), reactive oxygen species (ROS), and malondialdehyde (MDA) levels in the serum of tumor mice. TMQ treatment also increased the protein expression of Bax, Caspase-3, and Beclin-1 in tumor tissues. In contrast, the bcl-2, PI3K, Ki67, ULK1, and mTOR protein levels were suppressed by TMQ. Protein assay analyses reemphasized the superior antitumor effect of TMQ over TM. These cumulative findings demonstrated that the mechanism of action of TMQ was closely related to the activation of transcriptional misregulation in the cancer pathway that inhibited the cholinergic synaptic, AMPK, and PI3K/Akt/mTOR signaling pathways.

**Conclusion:**

This study demonstrated that fermentation increased the active ingredient contents and antitumor effects of *T. media*. In addition, post-fermentation TMQ was superior to TM as a herbal medicine for NSCLC treatment.

## 1 Introduction

Lung cancer is the most lethal cancer in China, and the types include non-small cell lung cancer (NSCLC) and small cell lung cancer. NSCLC accounts for approximately 80% of lung cancer cases (Lei et al., 2024). The primary treatment options for lung cancer include surgery, radiotherapy, and chemotherapy (Zheng et al., 2023). However, all these treatment options have certain adverse reactions or side effects (Housman et al., 2014; Bukowski et al., 2020), while traditional Chinese medicine (TCM) has become a research focus for treating lung cancer diseases because of its safe and effective advantages (Niu et al., 2024; Arjsri et al., 2023). Therefore, Taxus monodorous medicine, alone or in combination with other TCMs, is often used for the clinical treatment of lung cancer (Ma et al., 2020).

*Taxus media* (*Taxus media*) is slightly sweet, bitter, and belongs to the lung, stomach, and large intestine. It possesses efficacy in detoxifying and dispersing toxins, invigorating collaterals, relieving pain, inducing diuresis, and subduing swellings (Lei and Zhang, 2023, p. 172–179). *Taxus media* is also known as yew and was first mentioned by Li SZ in his book “De Materia Medica” as a treatment for colds and cholera (Wang et al., 2012). *Taxus media* are abundant in terpenes, phenols, lignans, and polysaccharides (Li et al., 2017; Zhou et al., 2019). The plant’s multiple active ingredients, exhibit various medicinal properties, such as an antitumor, antipyretic, analgesic, immune-regulating, and anti-inflammatory properties (Wang and Huang, 2018). Taxanes and flavonoids are the principal active phytoconstituents in Taxus media, and these can treat a variety of tumors, such as lung cancer, liver cancer, and ovarian cancer (Zhu and Chen, 2019; Sharma and Garg, 2015). Therefore, some Taxus plants and the Fuzheng Quxie decoction are used in hospitals as clinical applications for the treatment of lung cancer (Wang et al., 2007).

Lung cancer, belongs to the category of “lung stagnation” in Chinese medicine, and is primarily characterized by deficiency, toxicity, and stasis, i.e., internal deficiency in the body, invasion of external evils leading to an abnormal flow of fluids in the body, and blockage of qi and poor blood circulation, that in turn leads to stagnation of qi and blood stasis by blocking the lungs (Tang et al., 2016). The anti-cancer and anti-inflammatory effects of *Taxus* plants depend on their efficacy of detoxification, dissipation of stagnation, and in the induction of diuresis and swelling (Lou et al., 2015; Sharma and Garg, 2015; Dai et al., 2022). Studies have shown that the methanol extract, ethanol extract, and aqueous extract of Taxus plants can inhibit the proliferation of tumor cells after treatment. Another clinical study showed that the ratio of CD3^+^ and CD4^+^ cells and the CD4+/CD8+ ratio of non-small cell lung cancer patients increased after administering a *Taxus* decoction, suggesting that *Taxus* may boost the immunity of patients, thus prolonging their survival time (Ma et al., 2020). However, the precise mechanism through which it combats non-small cell lung cancer remains to be fully investigated.

Quji is a TCM with fermentation characteristics, that apparently originated more than 3,000 years ago. Quji is a solid preparation made by mixing a powder of the medicine and flour, maintaining an appropriate temperature and humidity, and allowing it to ferment naturally, or a preparation made by mixing fermented medicinal materials with other medicinal materials (Gong, 2016, p. 416) After fermentation, the chemical composition of traditional Chinese medicines will change. Fermentation and processing can increase the concentrations of active components in Chinese herbal medicines. In addition, macromolecular substances that are difficult to be absorbed by the human body will be converted into small molecules by microorganisms during the fermentation process. Moreover, the fermentation process may produce new compounds that may enhance the efficacy of the drug.

**FIGURE 1:**
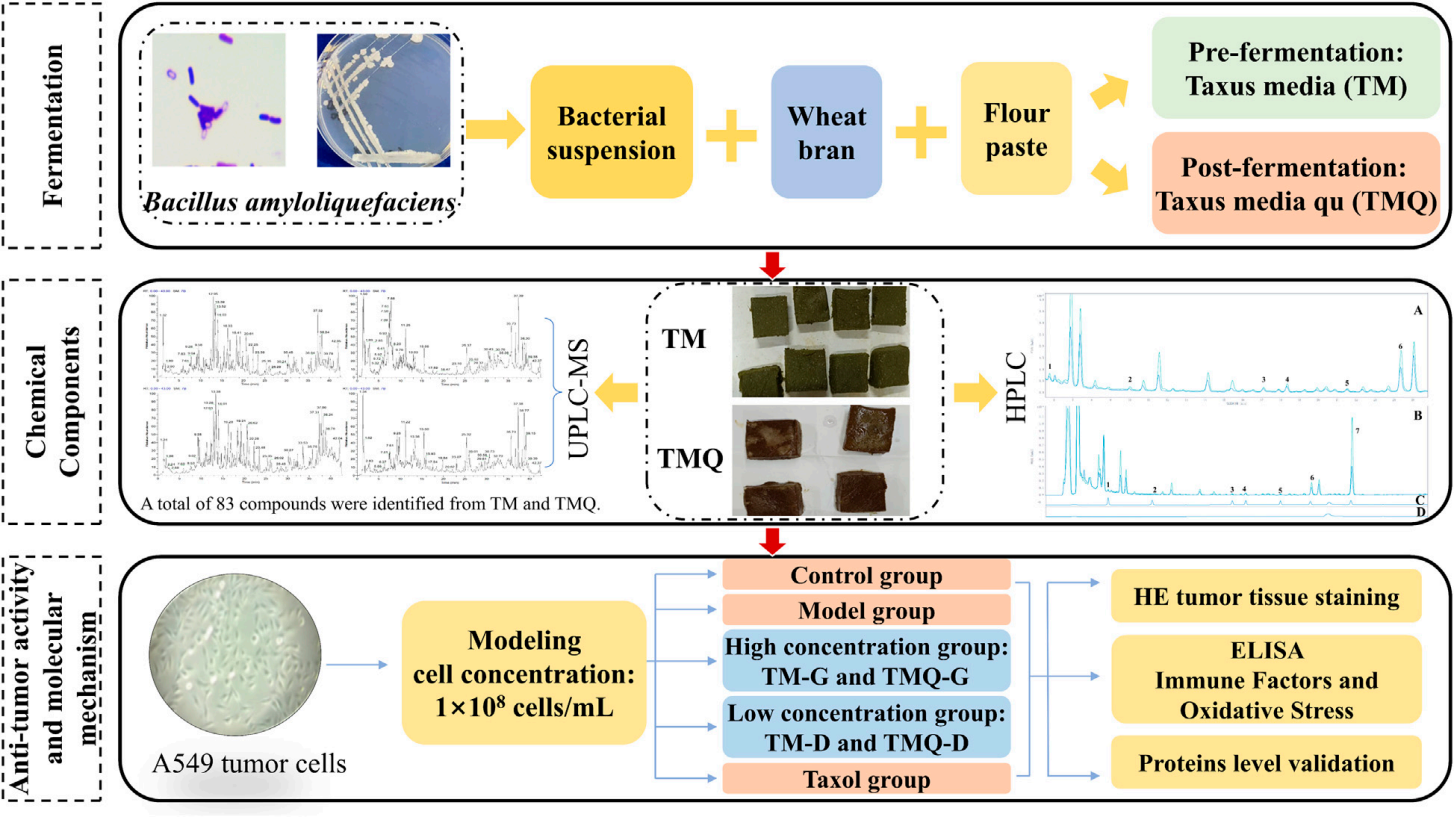
Schematic diagram of the technical process.

In this study, the fermentation method was used for the *T. media*. In addition, the pre-fermentation *T. media* (TM) and post-fermentation *T. media* qu (TMQ) were prepared. The antitumor effects of TM and TMQ were then compared using an animal model of the A549 tumor mice, and the mechanism of action of TM and TMQ against NSCLC was elucidated. The results showed that TMQ activated modulating immune factors, and oxidative stress, activated the cancer transcriptional deregulation pathways, inhibited the cholinergic synapses, the AMPK and PI3K/Akt/mTOR signaling pathways, thus exhibiting antitumor activity. These findings demonstrated that fermentation enhanced the efficacy of TM and TMQ in the treatment of NSCLC and provided a pharmacological rationale for the application of TMQ in NSCLC therapy. The technical flow chart design of our study is shown in Figure 1.

## 2 Methods and materials

### 2.1 Plant material and chemical reagents

Six to ten year-old *T. media* branches and leaves were provided from the Zhiyuan planting base (Zhiyuan Biotechnology Co., Ltd., Pingdingshan, China). The fresh plant samples were dried in an oven at 60°C and powdered for later use. The flour was purchased from the Jinsha River Flour Industry (Jinsha River Flour Industry Co., Ltd., Hebei, China). The wheat bran was purchased from the New Garden Agricultural Products Industry (New Garden Agricultural Products Industry Co., Ltd., Nanyang, China).

### 2.2 Preparation of TM and TMQ

A thin batter of *T. media* was boiled and placed at room temperature. A fine powder of *T. media* and wheat bran was mixed with the above, and a certain amount of bacterial suspension was added to soften the materials so it could easily be kneaded into balls by hand and dispersed upon tossing. Squares of a uniform size and thickness were then made, and some of them were dried in a drying oven at 60°C as the pre-fermentation samples (0 h sample or the TM sample). The rest were placed under a constant temperature and humidity (HMS-250, Tianjin Leiboterry Equipment Co., Ltd., Tianjin, China) fermentation box at a temperature of 32.7°C, humidity of 75%, and fermented for 60 h as the post-fermentation samples (TMQ samples). These were then dried and stored in a refrigerator at 4°C.

### 2.3 Chemical composition analysis of TM and TMQ

#### 2.3.1 Sample extraction

Each powder sample was accurately weighed to 1 g, placed in a tapered bottle, and mixed with 50 mL of methanol (Thermo Fisher Scientific China Co., Ltd, Shanghai, China). All samples were then weighed together for the total weight. Each sample was then extracted at 30°C for 30 min in an ultrasonic bath (power: 500 W; frequency: 40 kHz). The samples were then cooled it to room temperature, the lost weight was compensated using methanol, they were then shaken well. The supernatant was filtered, and 50 mL of methanol was added for a repeated extraction. The supernatant was extracted and combined with the filtrate. The filtrate was evaporated to dryness in a double-row six-hole instrument water bath (Shanghai ShuLi Instrument Co., Ltd., Shanghai, China) at 60°C, dissolved in methanol at a fixed volume of 10 mL, filtered through a 0.22 μm microporous membrane, placed in 1 mL of a solution in a 4°C centrifuge concentrator, concentrated in a vacuum for 5 h to dry, redissolved in 1 mL of methanol, and centrifuged using a high-speed refrigerated centrifuge. The supernatant was separated and filtered through a 0.22 μm microporous membrane.

#### 2.3.2 HPLC analysis

Qualitative and quantitative analyses were then performed on a Waters Symmetry C18 column (4.6 mm × 250 mm, 5 μm) using an Agilent 1260 high performance liquid chromatograph. The mobile phase were solution A (methanol) and solution B (0.1% phosphoric acid aqueous solution). The gradient elution procedure was as follows: 0–15 min, 65%–45% B; 15–22.5 min, 45%–30% B; 22.5–32.5 min, 30%–10% B; 32.5–35 min, and 10%–65% B. The flow rate, injection volume, and column temperature were 1 mL/min, 10 μL, and 30°C, respectively. The diode array detection wavelength was 232 nm. A qualitative analysis based on the peak area and retention time of the mixed standard compounds compared to sample peaks was performed. Appropriate amounts of 10-deacetylbaccatin III (10-DAB), baccatin III (DAB), cephalomannine, taxol, 7-epitaxol, ginkgetin and sciadopitysin were accurately weighed and added to methanol to prepare the concentrations of 0.6067 mg/mL, 0.2800 mg/mL, 0.2500 mg/mL, 0.1633 mg/mL, 0.0667 mg/mL, 0.3800 mg/mL, and 0.4400 mg/mL mixed control solutions. A six-point curve was constructed, and the regression equations and *R* ^2^ values were calculated for each standard.

#### 2.3.3 UHPLC-Q-qrbitrap HRMS analysis

The compounds in TM and TMQ were qualitatively identified using the Ultimate 3000-Orbitrap Exploris 240 LMS with Hypersil GOLD (100 × 2.1 mm, 1.9 μm) with a mobile phase flow rate of 0.2 mL/min. Aqueous formic acid (0.1%) was used as solvent A, and pure acetonitrile was used as solvent B. A gradient elution was applied under the following conditions: mobile phase A was kept at a slope of 95% for 0–0.5 min of the run, then decreased to 75% over 8 min, 50% over 24 min, 5% over 38 min, 5% over 40 min, 95% over 40.1 min, then kept at 95% until the end of the run. The volume of the sample analyzed was 4 μL. The scanning range for the positive and negative ion detection modes, and spray voltages were m/z 100–1200, and 3.5–3.0 kV, respectively. The sheath gas flow rate, and auxiliary gas flow rate were 25 arbitrary units (arb) and 10 arb, respectively, at 350°C for both the auxiliary and ion transfer tubes. Xcalibur 3.0 software, combined with the reference products and online databases such as PubChem, was used to analyze the mass spectrum data. The parent ions and fragment ions of the compounds were accurately compared, and the compounds in the samples before and after the fermentation of *T. media* were identified.

### 2.4 *In vitro* studies

#### 2.4.1 Cell line and cell culture

We obtained A549 cell lines, which are human pancreatic cancer cells, from the Service Biotechnology Company (Service Biotechnology Co., Ltd., Wuhan, China). The A549 cells were cultured in an RPMI1640 medium (Solar Biotechnology Technology Co., Ltd., Beijing, China) with a sterile incubator at 37°C with 5% CO_2_. Cells were resuscitated and passaged two to three times for the next step of the analysis when the cells were viable.

#### 2.4.2 Cell inhibition assay

The concentration of A549 cells was adjusted to 25,000 cells/mL and added to 96-well plates at 200 μL per well. They were then cultured in a 5% CO_2_ incubator at 37°C. The cells were cultured in a 5% CO_2_ incubator at 37°C and treated with medicine after the cells were attached to the wall. The preparation method of the drug was the same as that of the MS test solution. The methanol extract was concentrated to dry using a centrifugal concentrator, placed on an ultra-clean workbench for ultraviolet (UV) sterilization for 36 h, a complete medium was then added to dissolve, and the drug concentration was adjusted to 64 mg/mL.

The cell proliferation inhibition experiment dosing operation: The complete culture medium was used in a 5-mL Eppendorf (EP) tube for drug allocation. Each group was allocated 4 mL of the drug, and three compound pores were established. The corresponding final drug concentrations were 64, 32, 16, 8, 2, and 0.5 mg/mL. The 96-well plate medium was discarded, and 200 μL of the drug concentration medium of the corresponding group was added to each well. After the drug was administered for the specified time, the supernatant was discarded, 100 μL of a medium containing 10% CCK-8 was added, and incubation was continued for 1 h. The absorbance at 450 nm (optical density (OD) value) was measured using an enzyme marker, and the cell survival rate was calculated.

$$\text{Cell viability rate }\left(\%\right)=\left[A\;\left(\text{drug}\right)-A\;\left(\text{blank}\right)\right]/\;\left[A\;\left(\text{control}\right)-A\;\left(\text{blank}\right)\right]\times 100\%$$

Cell inhibition rate (%) = 1 - Cell viability rate.

A (drug): OD values of wells with cells, CCK-8 solution and drug solution.

A (control): OD value with cells, and CCK-8 solution but no drug solution.

A (blank): OD value without cells.

### 2.5 *In vivo* studies

#### 2.5.1 Animals and treatments

Male Balb/c-nu mice (18–20 g) were housed at the Animal Center with free access to food and water. The animals were acclimatized and fed for approximately 1 week prior to the experiments. All experiments were conducted in accordance with the guidelines of the Laboratory Animal Welfare Ethics Committee of the Department of Laboratory Animals, the Henan University of Chinese Medicine (Permit Number: DWLL202203316). A549 cells were cultured in large numbers, and cells were collected by centrifugation. Their concentration was adjusted to 1×10^8^ cells/mL, and each mouse was inoculated subcutaneously in the right anterior axilla with 0.2 mL. In approximately 4–7 days, a mung bean-sized hard mass was seen under the skin of the mice, and this was considered successful modeling. Tumor-bearing mice were randomly divided into six groups (*n* = 6): Model (M), TM high dose (TM-G), TM low dose (TM-D), TMQ high dose (TMQ-G), TMQ low dose (TMQ-D), and Taxol (T). The doses for TM and TMQ administration were calculated according to pharmacological experimental methodology, i.e., a clinical dose of 6 g/d for a 60 kg adult, with the low dose being two times the human clinical dose and the high dose being eight times the human clinical dose. The administered dose of the positive drug was equal to the human clinical dose. The high-dose group (9.872 g/kg) and the low-dose group (2.468 g/kg) were gavaged once a day, and the taxol group (7.62 mg/kg) was intraperitoneally injected once every 3 days. The mice were sacrificed on day 45.

#### 2.5.2 General status and tumor growth of the mice

The mice in each group were observed daily for diet, water intake, mental status, and activity. The body weights were measured every 4 days and the tumors were measured every 2 days. The tumor volume (mm^3^) of each mouse was calculated [V = a × b^2^ × 0.50, with a = the long side and b = the short side]. After 45 days of treatment, the mice were executed, and the tumor weights, tumor inhibition rates, and splenic indices were weighed and calculated. The tumor suppression rate = [tumor mass (M/g) – the tumor mass in treatment group (g)]/tumor mass (M/g) × 100%, spleen index = spleen mass (mg)/body mass (g).

#### 2.5.3 Evaluation of the serum oxidative stress and inflammatory factors

The levels of reactive oxygen species (ROS), malondialdehyde (MDA), glutathione (GSH), tumor necrosis factor-α (TNF-α), interferon-γ (IFN-γ) and interleukin-6 (IL-6) in the mouse serum were analyzed using enzyme-linked immunosorbent assay (ELISA) kits provided by Jiangsu Meimian Industrial Co., Ltd.

#### 2.5.4 Morphology of the tumor tissue section

The fresh tumor tissue was immersed with 4% paraformaldehyde, embedded, paraffin sectioned, and the cell morphology of the tumor tissue section was visualized using hematoxylin and eosin (H&E) staining.

#### 2.5.5 Immunohistochemical (IHC) staining

Tumor sections were processed using the primary antibodies Bax (GB114122), bcl-2 (GB114830), Caspase-3 (GB11532), and PI3K (GB11769) obtained from Servicebio Ltd. and then incubated with goat anti-rabbit IgG secondary antibodies. The specimens were stained with 3,3′-diaminobenzidine (DAB) and visualized under a microscope. The positive immunoreactivity revealed by DAB was brownish yellow. Images were quantitatively analyzed using Image Pro Plus software (6.0 version).

#### 2.5.6 Immunofluorescence (IF) staining

Six groups of tumor specimens were fixed, paraffin-embedded, and microtome-sectioned. These sections were incubated with primary antibodies targeting Ki67(GB121141, Servicebio, Wuhan, China), mTOR (GB111839, Servicebio, Wu-han, China), Beclin-1 (GB11228, Servicebio, Wuhan, China), and ULK1 (GB11580, Service-bio, Wuhan, China). The groups were then incubated with CY3-labeled goat anti-mouse IgG secondary antibody. 4′,6-diamidino-2-phenylindole (DAPI) was used to re-stain the nucleus, quench the tissue autofluorescence, seal the film, collect the image, and the positive immuno-reactivity was red. Images were quantitatively analyzed using Image Pro Plus software (6.0 version).

### 2.6 Statistical analysis

All data are presented as mean ± standard error of the mean (SEM). The statistical significance of the differences between groups was analyzed using a one-way analysis of variance (ANOVA). When analyzing the differences between groups, the least significant difference (LSD) multiple comparison method was used for the homogeneity of data variances, and the Dunnett’s T3 method was used for the heterogeneity of data variances. A value of *p* < 0.05 was considered statistically significant.

## 3 Results

### 3.1 Compositional studies of TM and TMQ

The regression equations for 10-DAB, DAB, cephalomannine, taxol, 7-epitaxol, ginkgetin, and sciadopitysin are shown in Supplementary Table S1. The contents of the selected taxanes and flavonoids in the samples are presented in Table 1. A quantitative analysis showed that the contents of 10-DAB, DAB, cephalomannine, taxol, 7-epitaxol, ginkgetin, and sci-adopitysin in TMQ were 23.25%, 20.42%, 27.74%, 25.58%, 22.10%, 18.15%, and 48.68% higher than in TM, respectively (Supplementary Figure S1). Chromatograms obtained using HPLC are presented in Supplementary Figure S2.

**TABLE 1:** Determination of 7 components in TM and TMQ (mg/g).

Sample	TM-1	TMQ-1	TM-2	TMQ-2	TM-3	TMQ-3	TM-4	TMQ-4	TM-5	TMQ-5
10-DAB	0.0918	0.1134	0.1001	0.1209	0.1114	0.1380	0.0959	0.1196	0.1020	0.1258
DAB	0.0647	0.0772	0.0596	0.0739	0.0651	0.0781	0.0713	0.0846	0.0670	0.0805
cephalomannine	0.1079	0.1391	0.1109	0.1397	0.0976	0.1262	0.0998	0.1279	0.1052	0.1329
taxol	0.1747	0.2225	0.1791	0.2214	0.1623	0.2044	0.1676	0.2086	0.1717	0.2172
7-epitaxol	0.0807	0.0994	0.0809	0.0995	0.0747	0.0909	0.0769	0.0925	0.0684	0.0837
ginkgetin	0.4723	0.5551	0.5897	0.7048	0.5310	0.6298	0.5626	0.6640	0.5667	0.6633
sciadopitysin	1.3530	2.0053	1.5918	2.3303	1.4198	2.1532	1.5018	2.2081	1.4472	2.1725

**FIGURE 2:**
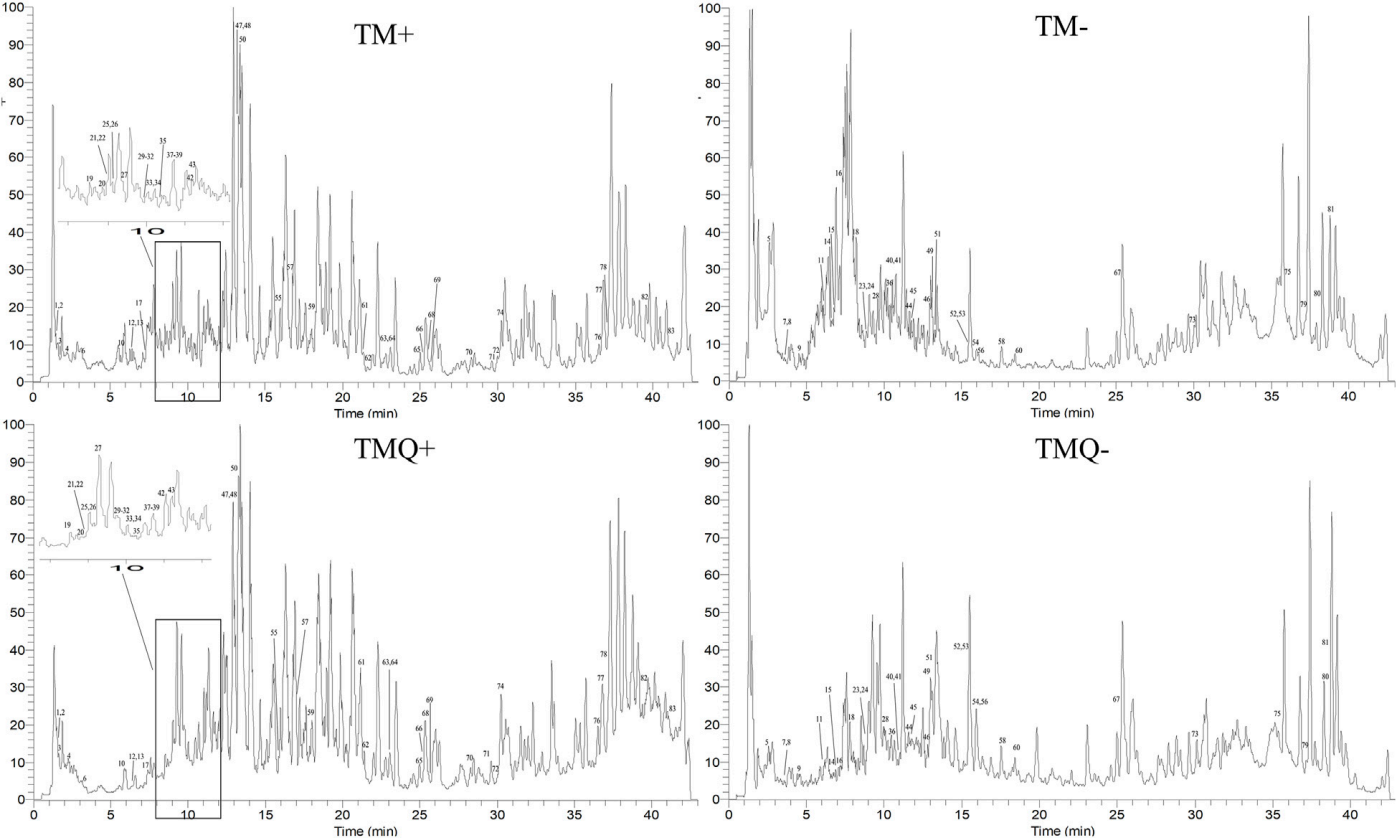
Total ion chromatograms (TICs) of TM and TMQ. (TM+) TIC of TM in positive ion mode; (TM-) TIC of TM in negative ion mode; (TMQ+) TIC of TMQ in positive ion mode; (TMQ-) TIC of TMQ in negative ion mode.

The positive and negative ion chromatograms of TM and TMQ are shown as Figure 2. The UHPLC-Q-Orbitrap HRMS identification results of the compounds in TM and TMQ are shown in Table 2. A total of 83 compounds, including two amino acids, one nucleoside, 21 organic acids, one phenol, one coumarin, 38 flavonoids, three lignans, 10 terpenes and six other types, were identified by reference materials, the literature, and the database of *T. media*. As shown in Figure 2 and Table 2, there were differences in the compound types and contents between TM and TMQ, with 81 compounds in TM and 79 compounds in TMQ. D-(−)-Quinic acid, taxchinin G, orientin, and prunin were detected only in TM, whereas isotaxiresinol, and neochlorogenic acid were detected only in TMQ.

**TABLE 2:** UHPLC-Q-Orbitrap HRMS-based identification of compounds in TM and TMQ.

No	Compound	Formula	Error/ppm	RT (min)	Adduct ions	Measured value	Peak intensity (m/z)	Source	Identification method	Ownership
1	Proline	C_5_H_9_NO_2_	−0.86	1.37	[M + H]^+^	116.0705	70.0651	a	Database, literature [21]	ALL
2	5-hydroxymethylfurfural	C_6_H_6_O_3_	0.00	1.44	[M + H]^+^	127.0390	53.0386、81.0334、109.0283	i	Database	ALL
3	Adenosine	C_10_H_13_N_5_O_4_	0.38	1.86	[M + H]^+^	268.1041	136.0617	b	Database, literature [22]	ALL
4	(2R, 3R)-5-ethoxy-2-[(ethoxyacetyl)amino]-3-methyl-5-oxopentanoic acid	C_12_H_21_NO_6_	0.36	2.29	[M + H]^+^	276.1443	212.1276、248.1486、258.1331、276.1437	c	Database	ALL
5	Vanillic acid	C_8_H_8_O_4_	0.00	2.43	[M − H]^−^	167.0350	123.0452、152.0115	c	Database	ALL
6	phenylalanine	C_9_H_11_NO_2_	0.60	3.26	[M + H]^+^	166.0863	79.0542、120.0807、131.0491、149.0595	c	Database	ALL
7	Isovanillic acid	C_8_H_8_O_4_	0.00	3.56	[M − H]^−^	167.0350	108.0216、123.0452、152.0113	c	Database	ALL
8	Gallic acid	C_7_H_6_O_5_	0.00	3.66	[M − H]^−^	169.0142	107.0139、125.0246	c	Database	ALL
9	protocatechuic acid	C_7_H_6_O_4_	0.65	4.48	[M − H] ^−^	153.0194	108.0217、109.0295	c	Database	ALL
10	DL-Tryptophan	C_11_H_12_N_2_O_2_	0.00	5.65	[M + H]^+^	205.0972	118.0650、170.0600、188.0706	a	Database	ALL
11	D-(−)-Quinic acid	C_7_H_12_O_6_	0.52	6.17	[M − H]^−^	191.0562	111.0088、127.0403	c	Database, literature [23]	TM
12	Epicatechin	C_15_H_14_O_6_	0.00	6.38	[M + H]^+^	291.0863	273.0763、291.0867	c	Database	ALL
13	4-Hydroxybenzoic acid	C_7_H_6_O_3_	0.72	6.40	[M + H]^+^	139.0390	77.0385、95.0491、121.0284、139.0390	c	Database	ALL
14	Neochlorogenic acid	C_16_H_18_O_9_	0.00	6.63	[M − H]^−^	353.0878	135.0451	c	Database	TMQ
15	6,7-Dihydroxycoumarin	C_9_H_6_O_4_	0.00	6.97	[M − H]^−^	177.0193	133.0295	d	Database	ALL
16	Caffeic acid	C_9_H_8_O_4_	−0.56	7.35	[M − H]^−^	179.0349	107.0502、135.0451	c	Database	ALL
17	Catechin	C_15_H_14_O_6_	0.34	7.47	[M + H]^+^	291.0864	273.0765、291.0864	e	Database	ALL
18	4-hydroxybenzaldehyde	C_7_H_6_O_2_	0.00	7.85	[M − H]^−^	121.0295	93.0345、108.0215、121.0295	c	Database	ALL
19	Guaijaverin	C_20_H_18_O_11_	0.00	8.52	[M + H]^+^	435.0924	73.0284、229.0495、303.0500、315.0501、435.0935	f	Database	ALL
20	5,6,7,8,3′,4′-Hexamethoxyflavone	C_21_H_22_O_8_	6.12	8.87	[M + Na]^+^	425.1232	123.0440	f	Database	ALL
21	Rutin	C_27_H_30_O_16_	0.33	8.96	[M + H]^+^	611.1609	303.0500、465.1051、611.1768	f	Database	ALL
22	Quercitrin	C_21_H_20_O_11_	2.23	9.02	[M + H]^+^	449.1088	303.0500、345.0641、413.0874、431.0969、449.1088	f	Database	ALL
23	Isohemiphloin	C_21_H_22_O_10_	0.46	9.11	[M − H]^−^	433.1142	119.0502、151.0036、177.0193、271.0613	f	Database	ALL
24	Cosmosiin	C_21_H_20_O_10_	0.00	9.13	[M − H]^−^	431.0983	269.0453	f	Database	ALL
25	Afzelin	C_21_H_20_O_10_	0.46	9.16	[M + H]^+^	433.1131	415.1026、433.1132	f	Database	ALL
26	Taxchinin G	C_28_H_40_O_11_	0.36	9.20	[M + H]^+^	553.2645	297.5018	i	Database	TM
27	Isoquercitrin	C_21_H_20_O_12_	0.22	9.43	[M + H]^+^	465.1029	85.0283、303.0499、304.0529	f	Database	ALL
28	Taxifolin	C_15_H_12_O_7_	−0.33	9.75	[M − H]^−^	303.0509	241.0505、285.0404	f	Database	ALL
29	Nicotiflorin	C_27_H_30_O_15_	0.17	9.81	[M + H]^+^	595.1658	287.0551、449.1074	f	Database	ALL
30	Luteolin-6-C-glucoside	C_21_H_20_O_11_	0.45	9.83	[M + H]^+^	449.1080	299.0545、329.0656	f	Database	ALL
31	Luteolin-7-O-glucoside	C_21_H_20_O_11_	0.89	9.88	[M + H]^+^	449.1082	153.0182、213.0544、287.0550	f	Database	ALL
32	Narcissoside	C_28_H_32_O_16_	−0.48	9.96	[M + H]^+^	625.1760	85.0283、285.0396、302.0426、317.0657、318.0692	f	Database	ALL
33	Kaempferol-3-O-galactoside	C_21_H_20_O_11_	0.89	10.20	[M + H]^+^	449.1082	153.0182、287.0551	f	Database	ALL
34	Orientin	C_21_H_20_O_11_	0.22	10.25	[M + H]^+^	449.1079	299.0556	f	Database	TM
35	Isorhamnetin-3-O-beta-D-Glucoside	C_22_H_22_O_12_	0.42	10.37	[M + H]^+^	479.1186	317.0657	f	Database	ALL
36	Isotaxiresinol	C_19_H_22_O_6_	−0.29	10.44	[M − H]^−^	345.1342	241.0502、267.0659、345.1342	g	Database	TMQ
37	Ecdysone	C_27_H_44_O_6_	0.64	10.59	[M + H]^+^	465.3213	429.2995、447.3095	i	Database, literature [10]	ALL
38	Prunin	C_21_H_22_O_10_	0.69	10.68	[M + H]^+^	435.1289	147.0440、153.0182、273.0760	f	Database	TM
39	Juglalin	C_20_H_18_O_10_	0.72	10.79	[M + H]^+^	419.0976	73.0284、153.0181、287.0550	f	Database	ALL
40	Eriodictyol-7-O-glucoside	C_21_H_22_O_11_	−0.22	11.05	[M − H]^−^	449.1088	135.0451、151.0036、287.0559	f	Database	ALL
41	Azelaic acid	C_9_H_16_O_4_	0.00	11.16	[M − H]^−^	187.0975	97.0658、125.0972、187.0975	c	Database	ALL
42	Aromadendrin	C_15_H_12_O_6_	0.00	11.22	[M + H]^+^	289.0707	243.0651、271.0601	f	Database, literature [10]	ALL
43	Schizandrin	C_24_H_32_O_7_	0.69	11.34	[M + H]^+^	433.2223	415.2124、433.2216	g	Database	ALL
44	3′-O-Methyltaxifolin	C_16_H_14_O_7_	0.00	11.50	[M − H]^−^	317.0667	137.0244、273.0767	f	Database	ALL
45	Kaempferol 7-O-β-D-Glucopyranoside	C_21_H_20_O_11_	0.00	11.59	[M − H]^−^	447.0932	284.0323、285.0404、447.0932	f	Database	ALL
46	Tricin-3-O-glucoside	C_26_H_36_O_9_	1.63	12.95	[M − H]^−^	491.2294	311.1651	f	Database, literature [24]	ALL
47	10-Deacetyl	C_29_H_36_O_10_	1.83	13.26	[M + H]^+^	545.2391	309.1487、327.1588、345.1698、363.1797	h	Database	ALL
48	10-Deacetylbaccatin III	C_29_H_36_O_10_	1.65	13.31	[M + H]^+^	545.2390	363.1808、345.1701	h	Standard, Database, literature [25]	ALL
49	Luteolin	C_15_H_10_O_6_	0.35	13.38	[M − H]^−^	285.0405	133.0295、151.0036、285.0404	f	Database	ALL
50	Quercetin	C_15_H_10_O_7_	0.33	13.40	[M + H]^+^	303.0500	229.0495、257.0445、303.0500	f	Database	ALL
51	Decanedioic acid	C_10_H_18_O_4_	0.00	13.48	[M − H]^−^	201.1132	139.1130、183.1028、201.1132	c	Database	ALL
52	Apigenin	C_15_H_10_O_5_	0.74	15.59	[M − H]^−^	269.0457	117.0346、118.0379、269.0455	f	Database	ALL
53	Naringetol	C_15_H_12_O_5_	−1.48	15.61	[M − H]^−^	271.0608	107.0138、119.0502、151.0036、187.0400	f	Database	ALL
54	Hesperetin	C_16_H_14_O_6_	−0.33	15.88	[M − H]^−^	301.0717	151.0037	f	Database	ALL
55	Kaempferol	C_15_H_10_O_6_	0.35	15.94	[M + H]^+^	287.0551	121.0283、153.0181、213.0544、241.0496、287.0550	f	Database	ALL
56	Fulgidic acid	C_18_H_32_O_5_	0.61	16.08	[M − H]^−^	327.2178	171.1027、229.1445、291.1970、327.2178	c	Database	ALL
57	Baccatin III	C_31_H_38_O_11_	1.53	16.98	[M + H]^+^	587.2495	105.0334、133.0648、327.1595、345.1708	h	Standard, Database, literature [25]	ALL
58	9,10,13-Trihydroxy-11-octadecenoic acid	C_18_H_34_O_5_	0.00	17.58	[M − H]^−^	329.2333	171.1026、229.1444、329.2333	c	Database	ALL
59	Amentoflavone	C_30_H_18_O_10_	0.00	18.37	[M + H]^+^	539.0972	539.0973、540.1004	f	Database	ALL
60	Dodecanedioic acid	C_12_H_22_O_4_	0.00	18.68	[M − H]^−^	229.1445	167.1440、211.1340、229.1445	c	Database	ALL
61	Daidzein	C_15_H_10_O_4_	0.00	21.28	[M + H]^+^	255.0652	255.0651	f	Database	ALL
62	Acacetin	C_16_H_12_O_5_	0.00	21.48	[M + H]^+^	285.0758	242.0574、270.0525、285.0757	f	Database	ALL
63	7-O Methyl amentoflavone	C_31_H_20_O_10_	0.36	23.16	[M + H]^+^	553.1131	417.0604、553.1126	f	Database, literature [10]	ALL
64	Deacetyltaxol	C_45_H_49_NO_13_	0.25	23.18	[M + H]^+^	812.3278	105.0334、122.0602、286.1082	h	Database	ALL
65	Cephalomannine	C_45_H_53_NO_14_	−0.48	25.08	[M + H]^+^	832.3535	264.1234、509.2179	h	Standard, Database	ALL
66	Ginkgetin	C_32_H_22_O_10_	−0.18	25.37	[M + H]^+^	567.1285	121.0283、431.0763	f	Standard, Database, literature [26]	ALL
67	Isoginkgetin	C_32_H_22_O_10_	−0.88	25.44	[M − H]^−^	565.1135	151.0037、533.0875、565.1136	f	Database	ALL
68	7-epi-10-deacetyl-taxol	C_45_H_49_NO_13_	0.00	25.60	[M + Na]^+^	834.3096	308.0892、549.2092	h	Database	ALL
69	Taxol	C_47_H_51_NO_14_	−0.47	25.85	[M + H]^+^	854.3378	105.03341、286.10767、122.05997	h	Standard, Database, literature [25]	ALL
70	7-Epitaxol	C_47_H_51_NO_14_	−0.23	28.31	[M + H]^+^	854.3380	286.1076、509.2172	h	Standard, Database, literature [25]	ALL
71	Aristolone	C_15_H_22_O	0.00	29.82	[M + H]^+^	219.1743	91.0541、203.1425	f	Database	ALL
72	4″-O- Methyl ginkgetin	C_33_H_24_O_10_	0.17	30.19	[M + H]^+^	581.1443	135.0440、549.1183	f	Database, literature [10]	ALL
73	Sciadopitysin	C_33_H_24_O_10_	−0.69	30.27	[M − H]^−^	579.1292	165.0193、388.0587、579.1292	f	Standard, Database, literature [27]	ALL
74	Sespendole	C_33_H_45_NO_4_	−4.42	30.47	[M + H]^+^	520.3398	104.1069、184.0733	h	Database, literature [10]	ALL
75	Linolenic acid	C_18_H_30_O_2_	0.00	35.81	[M − H]^−^	277.2173	277.2173	c	Database	ALL
76	(−)-Secoisolariciresinol	C_20_H_26_O_6_	2.75	36.54	[M + H]^+^	363.1812	79.0542、91.0542	g	Database	ALL
77	Palmitamide	C_16_H_33_NO	−0.39	36.83	[M + H]^+^	256.2634	88.0756、102.0913	i	Database, literature [28]	ALL
78	Beta-Sitosterol	C_29_H_50_O	0.96	37.24	[M + H]^+^	415.3938	57.0699、91.0541、119.0854	h	Database	ALL
79	Linoleic acid	C_18_H_32_O_2_	−0.36	37.39	[M − H]^−^	279.2328	279.2328	c	Database	ALL
80	Palmitic acid	C_16_H_32_O_2_	0.39	38.67	[M − H]^−^	255.2330	255.2329	c	Database	ALL
81	Oleic acid	C_18_H_34_O_2_	−0.36	39.10	[M − H]^−^	281.2485	281.2484	c	Database	ALL
82	Octadecanamide	C_18_H_37_NO	−0.35	39.57	[M + H]^+^	284.2947	71.0855、102.0913、284.2948、285.2982	i	Database, literature [28]	ALL
83	Pyrocatechol	C_6_H_6_O_2_	−0.90	40.87	[M + H]^+^	111.0439	65.0386、111.0439	i	Database	ALL

Note: a: amino acid; b: nucleoside; c: organic acid; d: coumarin; e: Phenol; f: flavonoids; g: lignans; h: terpenes; i: other. ALL represents the presence of the compound in both TM and TMQ. Database refers to the pubchem (https://pubchem.ncbi.nlm.nih.gov/).

### 3.2 Anticancer activity

**FIGURE 3:**
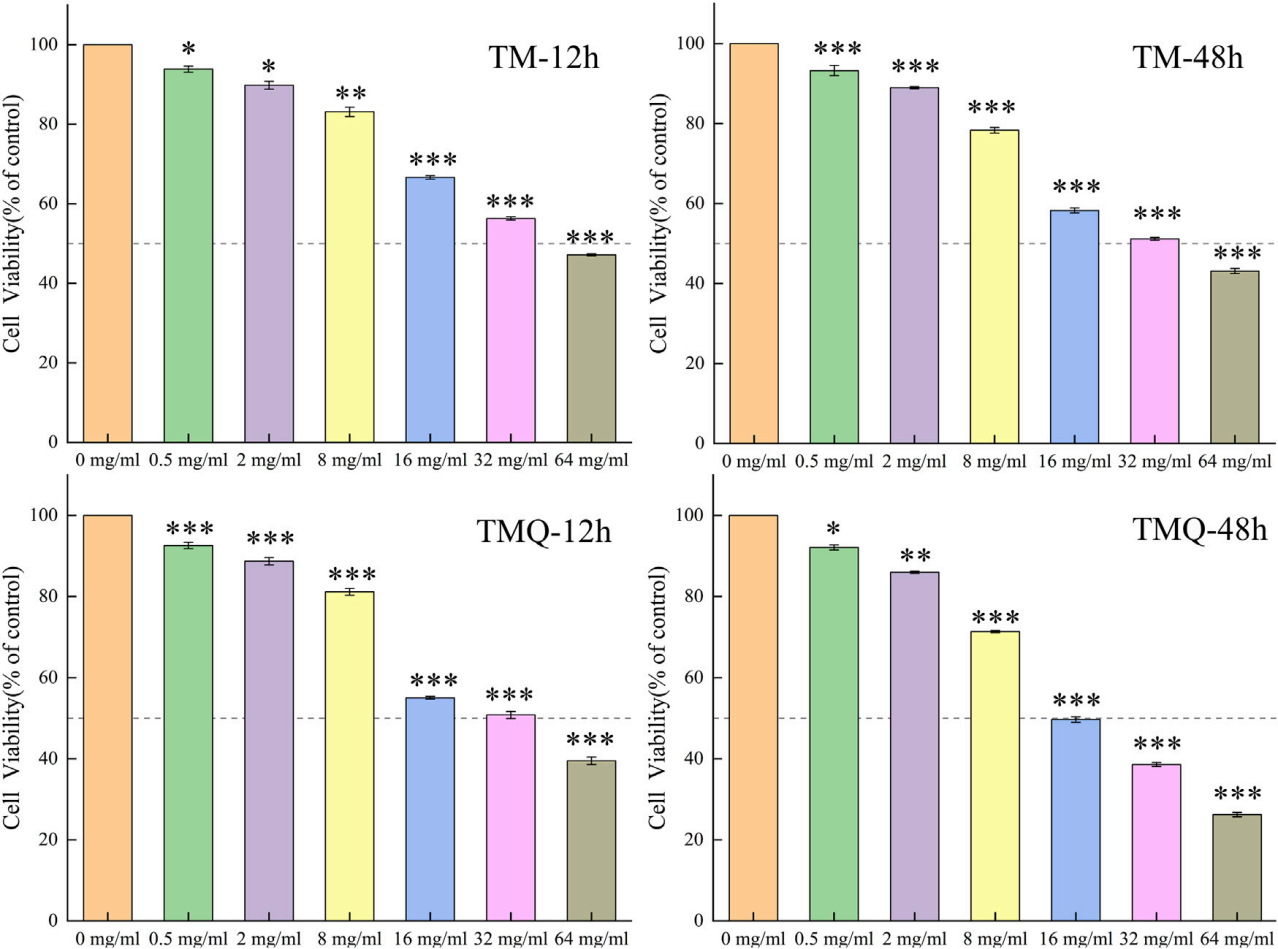
The effects of TM and TMQ on A549 cells viability were determined by CCK8 assay. The A549 cells were treated with 0–64 mg/mL of TM and 0–64 mg/mL of TMQ for 12, and 48 h. Data are presented as mean ± S.D. values of three experiments. *p < 0.05, **p < 0.01 and ***p < 0.001 compared to the control (0 mg/mL).

To evaluate the bioactivity of TM and TMQ, we performed a CCK8 assay using methanolic extracts. The results showed that TM had a cytotoxic effect on A549 cells, with a IC_50_ values of 37.87 mg/mL and 26.83 mg/mL after incubation for 12 h and 48 h, respectively. In contrast, the IC_50_ values of TMQ were even lower at 27.71 mg/mL and 19.43 mg/mL after 12 h and 48 h of incubation, respectively. The figures indicate that the cytotoxicity was higher than that of the TM. The antitumor abilities of TM and TMQ were evaluated based on the inhibitory rate of the drug on A549 lung cancer cells (Supplementary Figure S3). The inhibitory abilities of the TMQ samples in each drug group were better than those of TM (Figure 3). When the drug acted on A549 lung cancer cells for 12 h, there was no significant difference in the inhibitory ability between TM and TMQ of the drug group below 16 mg/mL. However, at 16 mg/mL and above, the inhibitory ability of the administration group was significantly higher than that of TM. When acting for 48 h, the inhibitory abilities of the six groups were significantly higher than those of TM.

### 3.3 Influence of TM and TMQ on the body weights and tumors in the tumor mice

**FIGURE 4:**
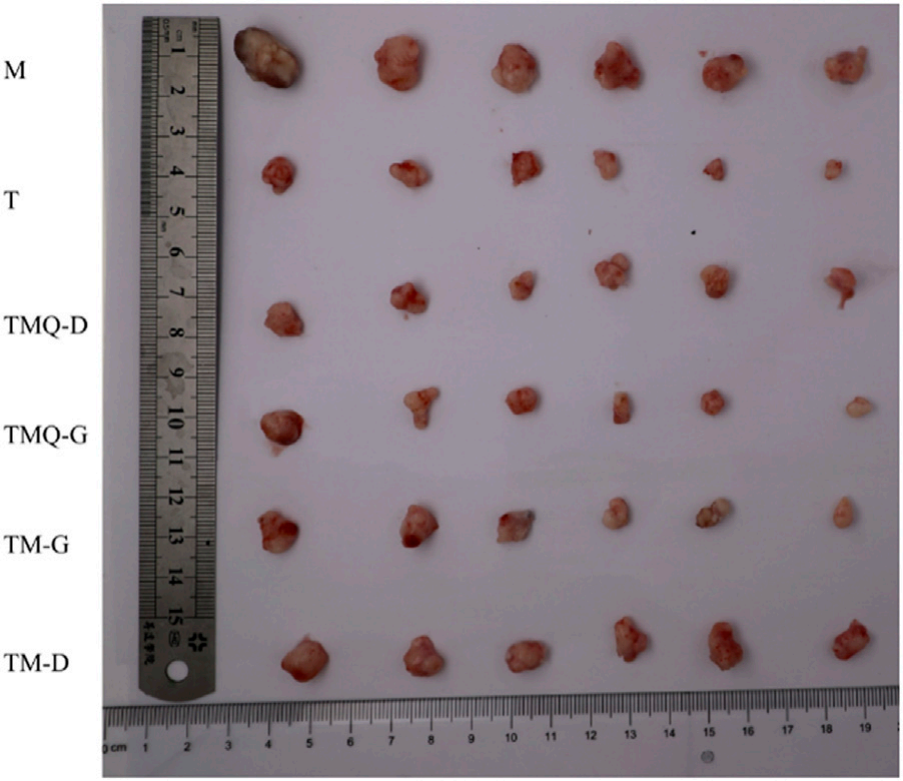
The effects of TM and TMQ on tumor tissue.

The results showed that the mice body weights in the model group were significantly higher than that in the administration groups, and the spleen indices were significantly lower than that in the administration groups. The tumor volumes and mass of the model group were the largest among the six groups of tumor-bearing mice, and all the administration groups effectively inhibited tumor growth (*p* < 0.05). The tumor volumes in the TMQ group were smaller than those in the TM group, while the tumor inhibition rate in the TMQ group was higher than that in the TM group (Figure 4). Moreover, after fermentation, the tumor inhibition rate of the TMQ-G group reached 57.24% (Table 3), which was very close to that of the positive drug group. The above results showed that the antitumor activity of TMQ was higher than that of TM, and the antitumor activity of the high-dose-group was stronger than that of the low-dose group.

**TABLE 3:** The result of body mass, splenic index, tumor volume, tumor mass, and tumor inhibition rate of mice in each group after drug administration.

Groups	Mice Weight(g)	Spleen index	Tumor Value (mm^2^)	Tumor Weight(g)	Tumor suppression rate (%)
CON	23.05 ± 0.49^ab^	3.41 ± 0.11^c^	—	—	—
M	23.41 ± 0.53^a^	2.42 ± 0.14^f^	782.70 ± 237.72^a^	0.81 ± 0.20^a^	—
T	22.78 ± 0.62^b^	3.75 ± 0.25^b^	233.95 ± 114.35^c^	0.34 ± 0.11^c^	57.37%
TMQ-G	22.11 ± 0.49^c^	4.42 ± 0.16^a^	234.87 ± 144.87^bc^	0.35 ± 0.10^c^	57.24%
TMQ-D	22.37 ± 0.35b^c^	3.21 ± 0.09^d^	295.58 ± 87.04^c^	0.44 ± 0.08^c^	45.86%
TM-G	22.22 ± 0.17^c^	4.33 ± 0.13^a^	288.03 ± 156.75^bc^	0.41 ± 0.14^c^	49.62%
TM-D	22.58 ± 0.33^bc^	3.04 ± 0.11^e^	445.25 ± 133.24^b^	0.59 ± 0.06^b^	27.60%

Note: CON, M, T, TMQ-G, TMQ-D, TM-G, and TM-D, denote normal group, model group, taxol positive drug group, high-dose group of TMQ, low-dose group of TMQ, high-dose group of TM, and low-dose group of TM, respectively. Different letters in the same column indicate a significant difference (*p* < 0.05) (*n* = 6).

### 3.4 Effects of TM and TMQ on tumor tissue in mice

**FIGURE 5:**
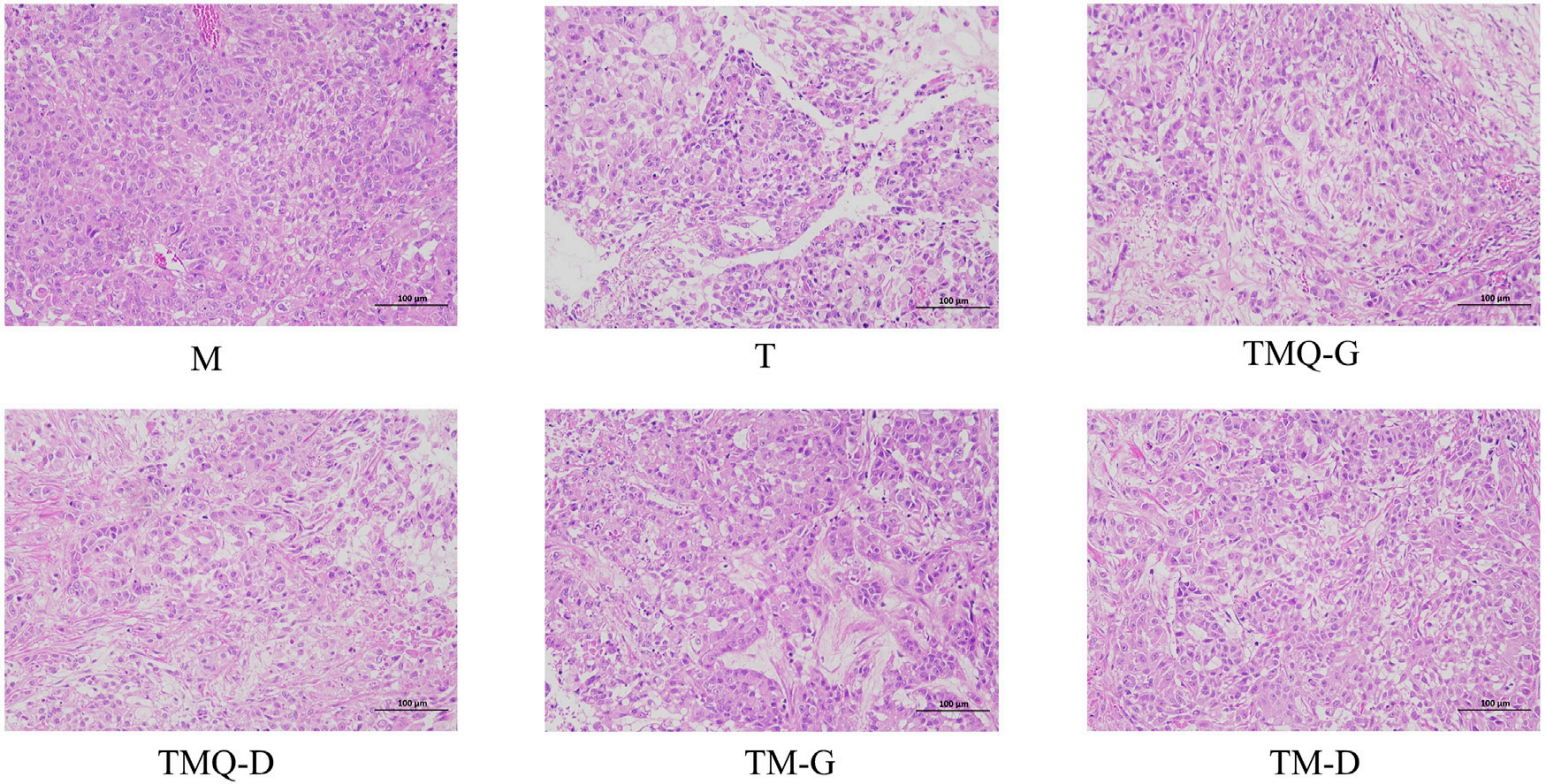
Effects of TM and TMQ on the histopathology of mouse tumor tissues (200×) (*n* = 3).

The results of the H&E staining showed that the tumor tissues of mice in the model group were characterized by abundant blood vessels, tight cell arrangements, irregular shapes, mostly round or short fusiform, sparse cytoplasm, unclear boundaries between cells, asymmetric chromosomes in the nucleus, and generally asymmetric division and multinucleation (Figure 5). In the same field of view, blood vessels and tumor cells were greatly reduced in the T group, with large cellular gaps in the tumor tissues and a large number of tumor cells with broken nuclei. The tumor tissue cell gap was larger and the number of tumor cells was reduced in the mice of each TCM group. Compared with TM, TMQ greatly increased the inhibition of tumor cell proliferation.

### 3.5 Effect of TM and TMQ on immune factors and oxidative stress in the mice serum

**FIGURE 6:**
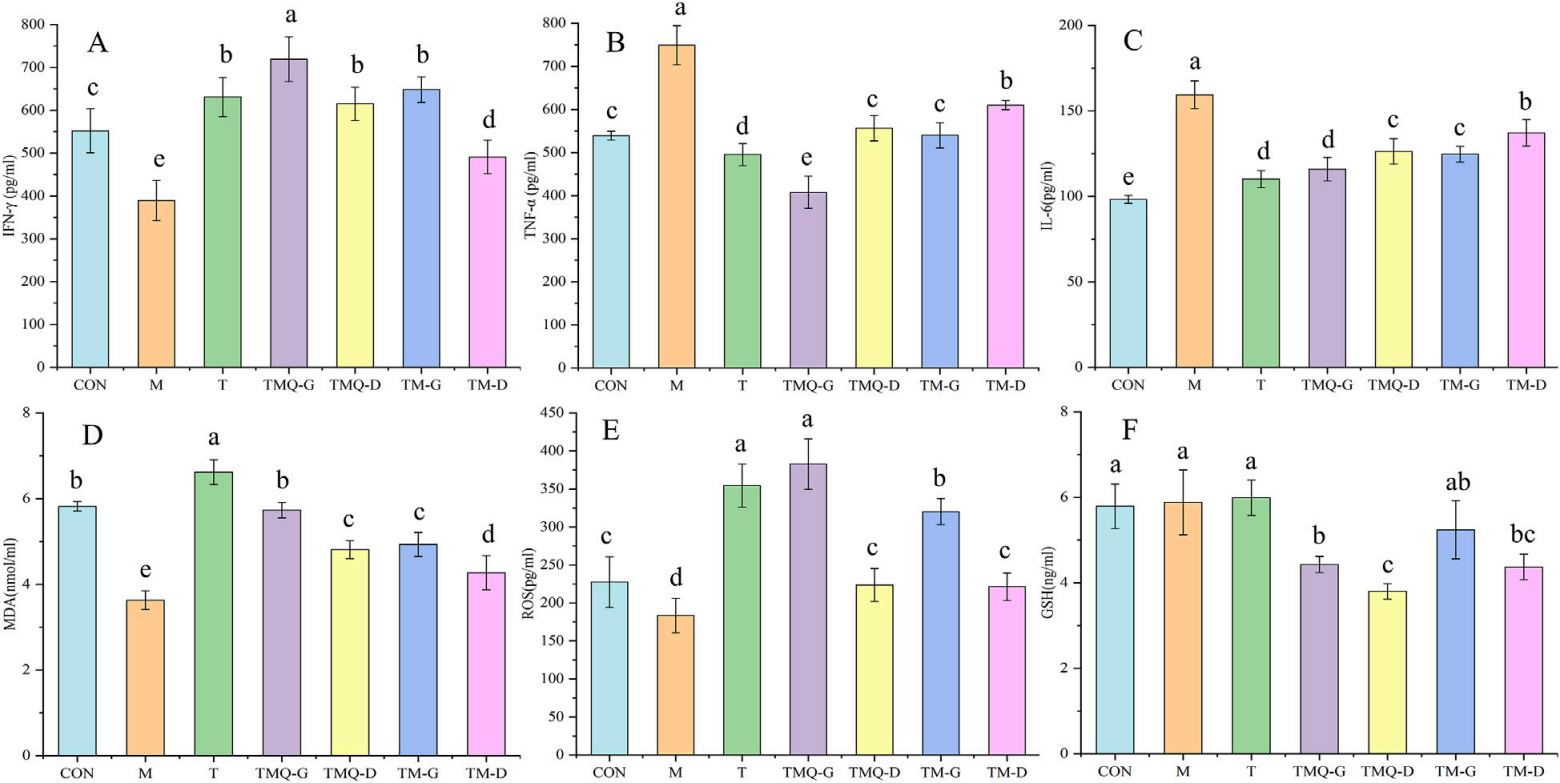
Results of immune factors and oxidative stress indexes. **(A)** IFN-γ, **(B)** TNF-α, **(C)** IL-6, **(D)** MDA, **(E)** ROS and **(F)** GSH were determined by ELISA. Different letters in the same column indicate a significant difference (*p* < 0.05) (*n* = 6).

Inflammation is closely related to cancer development and progression, and inflammation is primarily associated with changes in the levels of IL-6, TNF-α, and IFN-γ (Wang et al., 2024; Abidi et al., 2024). Additionally, inflammation and oxidative stress are two interrelated processes, and it has been shown that increasing ROS and MDA and decreasing GSH can help inhibit tumor cell proliferation (Wang et al., 2024). The serum levels of the above indicators in mice are shown in Figure 6. Compared to the M group, the expression of IFN-γ, ROS, and MDA in each Chinese medicine group was significantly upregulated (*p* < 0.05), while the expression of TNF-α, IL-6, and GSH significantly decreased, indicating that the four Chinese medicine groups could inhibit the proliferation of tumor cells by reducing inflammatory factors, regulating immune function, and promoting oxidative stress. In addition, IFN-γ, ROS, and MDA in the TMQ-G group were significantly higher than in the TM-G group, and TNF-α and IL-6 in the TMQ-G group were significantly lower than in the TM-G group (*p* < 0.05). IFN-γ and MDA in the TMQ-D group were significantly higher than in the TM-D group, and TNF-α and IL-6 were significantly lower than the levels prior to fermentation (*p* < 0.05), which again indicated that the antitumor ability of the TMQ samples was stronger than that of the TM samples.

### 3.6 Effects of TM and TMQ on the transcriptional misregulation in cancer pathway and tholinergic synapse pathway in A549-induced mice tumors

**FIGURE 7:**
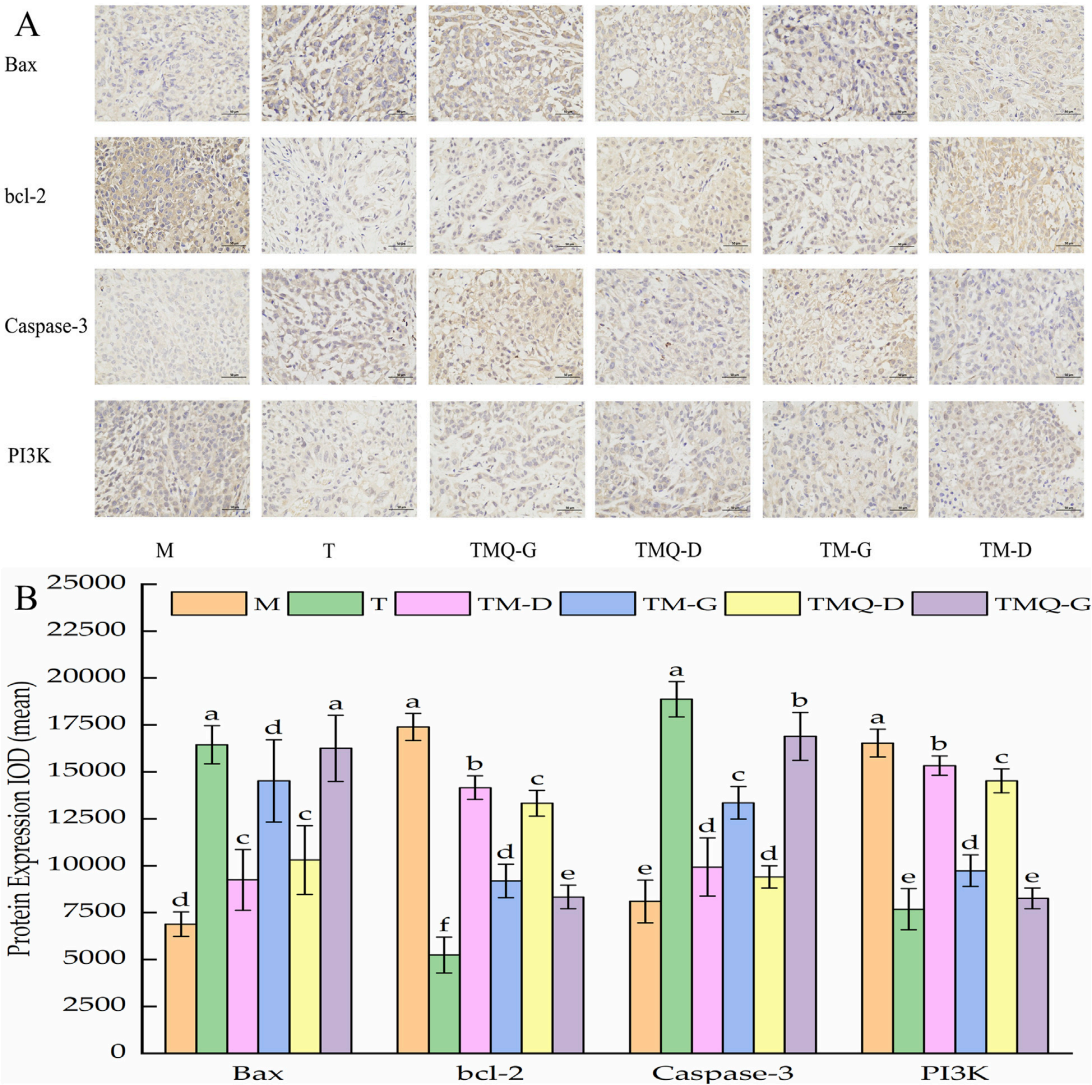
Immunohistochemical staining sections of Bax, bcl-2, Caspase-3 and PI3K in mouse tumor tissues **(A)** and their expression results **(B)**.

As shown in Figure 7, it was inferred that Bax and the caspase-3 protein expression levels were significantly higher (*p* < 0.05) and bcl-2 and the PI3K protein were significantly lower (*p* < 0.05) in the group treated with TM and TMQ than in the model group. Furthermore, the tumors of mice under same dose of mice treated with TMQ had considerably high (*p* < 0.05) expression levels of Bax compared with the TM group. Additionally, the expression levels of bcl-2 and the PI3K protein were significantly lower (*p* < 0.05) than those in the TM group.

### 3.7 Effects of TM and TMQ on the AMPK signaling pathway and PI3K/Akt/mTOR signaling pathway in A549-induced mice tumors

**FIGURE 8:**
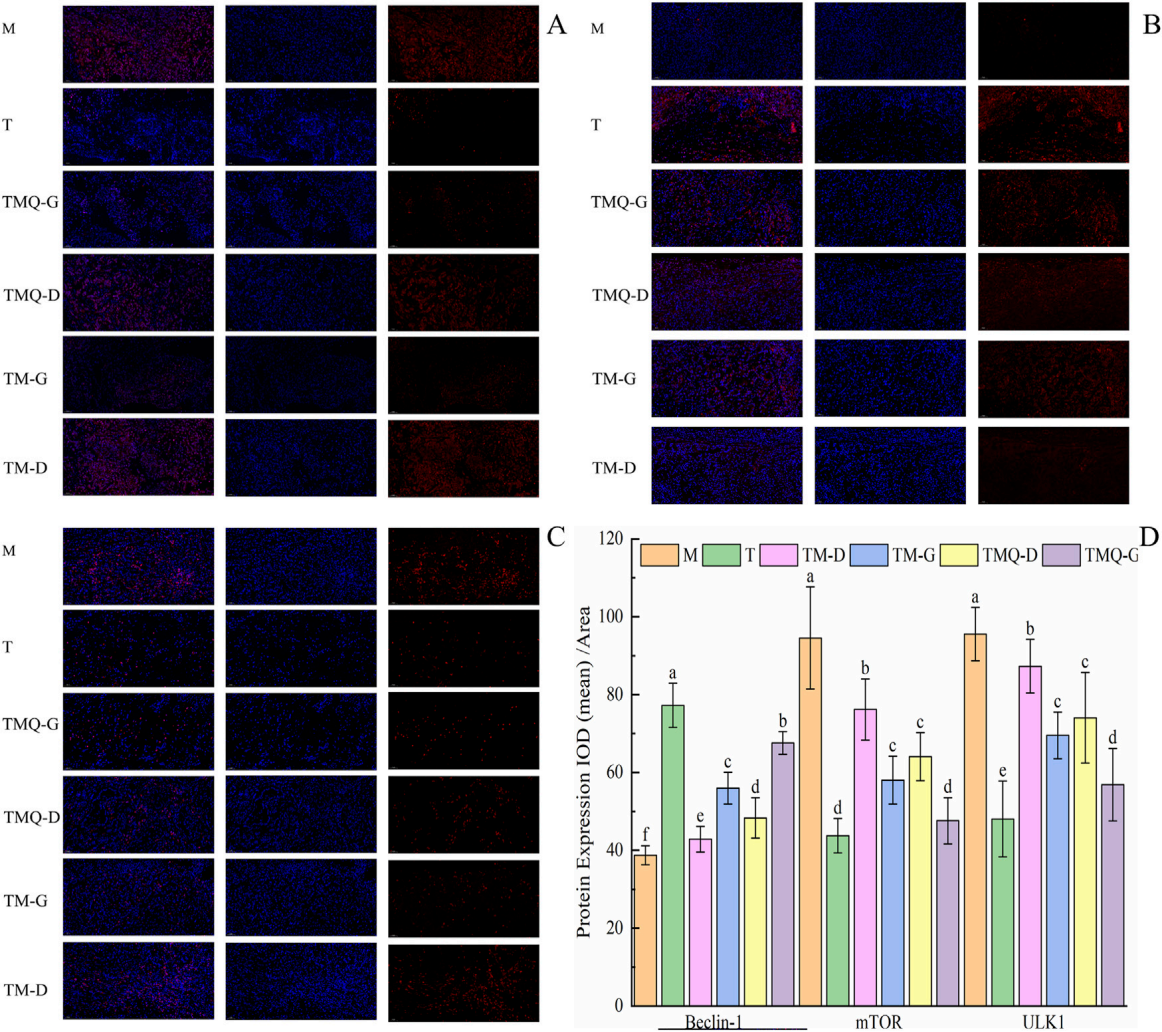
Expression of mTOR **(A)**, Beclin-1 **(B)**, ULK1 **(C)** and quantitative analysis **(D)** in mouse tumor tissues. ^a–f^ The different letters denote statistically significant differences (*p* < 0.05) in the data’s mean values between the ex-perimental groups (*n* = 3).

The results in Figure 8 show that the administration of TCM significantly reduced the expression levels of mTOR and the ULK1 proteins in the mice tumors (*p* < 0.05), but the levels were higher than those in the T group. In addition, TCM significantly increased the expression level of the Beclin-1 protein in mice tumors (*p* < 0.05), but the levels were lower than those in the T group. This was in contrast to the levels in the model groups.

### 3.8 Effects of TM and TMQ inhibition of cell proliferation in mice tumors

**FIGURE 9:**
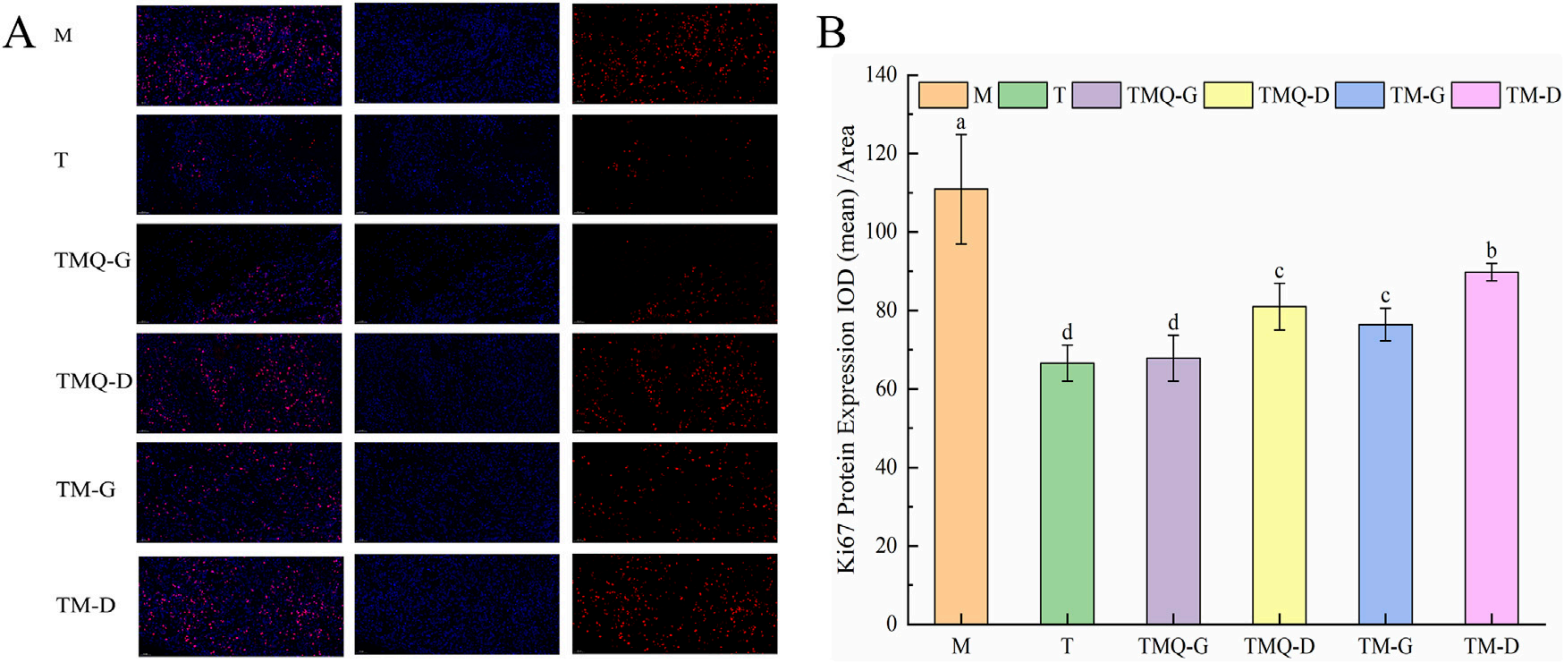
Expression of Ki67 **(A)** and quantitative analysis **(B)** in mouse tumor tissues. ^a–d^ The different letters denote statistically significant differences (*p* < 0.05) in the data’s mean values between the experimental groups (*n* = 3).

Figure 9 shows that the expression levels of the Ki67 protein in the mice tumors were significantly reduced in each administration group compared with the model group (*p* < 0.05). The T group showed the lowest Ki67 protein expression, followed by the TMQ-G group. Additionally, post-fermentation TMQ significantly reduced the expression level of the Ki67 protein in the mice tumors (*p* < 0.05).

## 4 Discussion

Lung cancer is one of the three major cancers and poses the greatest threat to life (de-Medeiros et al., 2024). Although many studies have been conducted on the treatment of lung cancer in recent decades, these methods have not significantly improved the survival rate of lung cancer patients (Lin et al., 2016). Many chemotherapy methods are expensive and unaffordable for patients and have serious adverse reactions and side effects that affect the quality of life of patients (Housman et al., 2014; Bukowski et al., 2020), Chinese herbal medicine is safe and effective, with little side effects, and has a moderate price; hence, it has gradually become a significant research subject for the treatment of lung cancer. Studies have shown that after solid fermentation, traditional Chinese medicine can not only inhibit the proliferation of tumor cells, but also kill tumor cells by improving immunity, and the inhibition effect after fermentation is stronger than that prior to fermentation (Chen et al., 2020; Liu et al., 2023; Zhao, 2021).

The objective of our study was to investigate the effects of fermentation on the chemical composition and antitumor activity of *T. media* and the molecular mechanism of its treatment of NSCLC. The enhancement of antitumor activity after fermentation cannot be separated from specific active components of TCM. Therefore, HPLC and UPLC-MS experiments were first conducted to study the effects of fermentation on the chemical components of *T. media*. The HPLC results showed that the contents of 10-DAB, DAB, cephalomannine, taxol, 7-epitaxol, ginkgetin, and sciadopitysin in TMQ were 23.25%, 20.42%, 27.74%, 25.58%, 22.10%, 18.15%, and 48.68% higher than in TM, respectively. A total of 83 compounds were identified by UHPLC-Q-Qrbitrap HRMS, most of which were important terpenoids, flavonoids and organic acids. The composition study showed that the types of compounds in *T. media* were basically the same before and after fermentation, and the difference was primarily in content. The contents of antitumor components in TMQ were higher than that in TM; therefore, we speculate that its antitumor effect was also better than TM. We also used A549 lung cancer cells for preliminary verification of our speculation. The results of the CCK8 experiment confirmed that the inhibition rate of high concentration TMQ was 73.78%, which was significantly higher than that of the TM sample. We confirmed that this was consistent with the results obtained by Shu Q and colleagues (Shu et al., 2014), whose inhibition rate was the highest at 42.23%. In this study, the inhibition rate of *T. media* on A549 lung cancer cells was 60.49%, which may have been due to the longer effect of the experimental drug on cells.

Subsequently, *in vivo* experiments were conducted using mouse models to explore the antitumor activities of TMQ and TM and their molecular mechanisms in treating non-small cell lung cancer. The mice were divided into a control group, a model group, a positive drug group and TMQ and TM groups with different doses. We measured the body mass and tumor related parameters in the mice. The results of this study showed that, with the administration of TM and TMQ, the body weights, tumor volumes, and mass were lower, and the splenic indices were higher than in M group, indicating that TM and TMQ promoted the development of the mice spleens, improved the immune ability of the mice, and inhibited the proliferation of tumor cells. The inhibition rate of the TMQ-G group (57.24%) was close to that of the T group (57.37%). The diet and water consumption of the mice in the TMQ-G group were higher than that of the T group, and the vitality of the mice was more vigorous, suggesting that the TMQ drug better improved the quality of life of the mice. We then used an ELISA kit to detect the inflammatory factors and markers of oxidative stress in the serum.

Studies have confirmed that chronic inflammation, such as in pneumonia, hepatitis, and colitis, may promote the development of cancers such as lung, liver, and colon cancer (Remila et al., 2015; Metwally et al., 2024; Tan et al., 2024). IFN-γ is a cellular interferon that can regulate immune function and inhibit tumor cell proliferation, TNF-α is related to inflammatory response and immune response (Lei et al., 2024), and IL-6 is the most central inflammatory factor that links inflammation and tumors (Liu et al., 2024). The results showed that the IFN-γ, TNF-α, and IL-6 levels significantly increased in all administration groups (*p* < 0.05) vis-à-vis the M group. The effects of the TMQ-G group and T group on the levels of inflammatory factors were basically the same. Studies have shown that reducing TNF-α and IL-6 levels and increasing IFN-γ can help inhibit inflammation and tumor cell proliferation (Situmorang et al., 2024). In addition, oxidative stress plays an important role in cancer occurrence and development. Excessive ROS can cause oxidative stress damage and induce apoptosis in tumor cells. GSH can scavenge free oxygen, and GSH depletion can induce tumor cell apoptosis (Abidi et al., 2024; Özbeyli et al., 2017). MDA is a product of lipid peroxidation, and it can cause lipid peroxidation in cell membranes and apoptosis (Shokrian-Zeini et al., 2024; Xie et al., 2022). Compared with the M group, the TMQ-G group exhibited significantly increased, ROS and MDA levels, while the GSH level in the TMQ-G group significantly decreased (*p* < 0.05). Increases in the ROS and MDA levels and decreases in the GSH level help to aggravate the oxidative damage to cell membranes, and this leads to impaired functions of tumor cells and inhibits tumor growth. Compared with the same dose prior to fermentation, the IFN-γ, ROS, and MDA levels significantly increased, and the TNF-α and IL-6 levels significantly decreased after fermentation (*p* < 0.05). In conclusion, TMQ and TM inhibited tumor proliferation by regulating the related indices of inflammatory factors and oxidative stress, and TMQ had a significantly stronger inhibitory ability on tumor proliferation than TM.

To investigate the mechanism of TMQ and TM in inhibiting non-small cell lung cancer, we performed a tumor biopsy analysis. The apoptosis of tumor cells is closely related to Bax and bcl-2, and Bax and bcl-2 are proapoptotic factors and antiapoptotic factors. Caspase-3 is also an apoptotic factor (Wu et al., 2014). These three factors jointly regulate the transcriptional dysregulation of tumor pathways. The expression of Bax and caspase-3 significantly increased in the four Chinese medicine groups compared with the M group, while the expression of bcl-2 significantly decreased (*p* < 0.05). In addition, the expression of Bax and the caspase-3 protein in the TMQ-G group was significantly higher than that in the TM-G group, and the expression of bcl-2 was significantly lower than that in the TM-G group (*p* < 0.05). These data suggested that TMQ may induce tumor cell apoptosis by activating the tumor transcriptional dysregulation pathway. PI3K and bcl-2 proteins are located downstream of the cholinergic synaptic pathway and are closely related to cell proliferation and differentiation. PI3K and ULK1 proteins are located downstream of the AMPK signaling pathway. Compared with the M group, the expression levels of bcl-2 and the PI3K and ULK1 proteins in the TMQ group and TM group significantly decreased (*p* < 0.05), indicating that the Chinese medicine inhibited the cholinergic synaptic pathway and AMPK signaling pathway by downregulating the PI3K, Bcl-2, and ULK1 proteins and thus inhibiting the proliferation of tumor cells. According to our results, TMQ showed more potent antitumor activity than TM. The occurrence and development of tumors are closely related to tumor cell proliferation, apoptosis, and autophagy (Li et al., 2021; Li et al., 2024). The PI3K/AKT/mTOR pathway is one of the most common dysregulated pathways in cancer (Janku et al., 2018), and the PI3K/AKT/mTOR signaling pathway is closely related to tumor cell proliferation and angiogenesis. Therefore, we examined the levels of beclin-1 and mTOR proteins. The results showed that TMQ increased the expression of the beclin-1 protein and activated autophagy. It also downregulates PI3K and mTOR proteins (Rossetti et al., 2024), inhibits the PI3K/Akt/mTOR signaling pathway, and enhances autophagy. In addition, Ki67 is closely related to cell mitosis, and higher expression indicates more active cell proliferation (Groen-van-Schooten et al., 2024). The expression of the Ki67 protein in the TMQ group was significantly lower than that in the TM and M groups (*p* < 0.05). In summary, these data indicated that TMQ enhanced tumor autophagy and effectively inhibited tumor growth in mice by activating the cancer transcriptional dysregulation pathway, and inhibiting the cholinergic synaptic pathway, the AMPK signaling pathway and the PI3K/Akt/mTOR signaling pathway, thus playing a role in the treatment of non-small cell lung cancer. The therapeutic effect of TMQ on non-small cell lung cancer was significantly stronger than that of TM. The limitations of our study require further exploration regarding the mechanism by which TMQ simultaneously activates the tumor transcriptional dysregulation pathway, and inhibits the cholinergic synaptic pathway, the AMPK signaling pathway, and the PI3K/Akt/mTOR signaling pathway, and the clinical relevance of these mechanisms. In addition, safety studies on the clinical use of TMQ are insufficient and further *in vitro* and *in vivo* studies are necessary.

## 5 Conclusion

This study confirmed that TM and TMQ had positive effects on A549 cell-induced lung cancer. TMQ showed superior therapeutic effects to TM in non-small cell lung cancer, including an increased splenic index, IFN-γ, ROS, and MDA levels, and decreased TNF-α and IL-6 levels. In addition, the therapeutic mechanisms of TM and TMQ were related to the activation of transcriptional misregulation in the cancer pathway, inhibition of the cholinergic synapse pathway, the AMPK signaling pathway and the PI3K/Akt/mTOR signaling pathway, and inhibition of tumor cell proliferation. This study demonstrated that fermentation enhanced the efficacy of the TCMs in the treatment of NSCLC, elucidated a portion of the molecular mechanism of TM and TMQ in the treatment of NSCLC, and provided pharmacological data for the application of TMQ in the treatment of NSCLC.

## Data Availability

The original contributions presented in the study are included in the article/Supplementary Material, further inquiries can be directed to the corresponding author.

## Abbreviations

NSCLC, Non-small cell lung cancer; TCM, traditional Chinese medicine; TM, pre-fermentation Taxus media; TMQ, post-fermentation Taxus media qu; TNF-α, tumor necrosis factor-α; IL-6, interleukin-6; GSH, Glutathione; IFN-γ, interferon-γ; ROS, reactive oxygen species; MDA, malondialdehyde; T. media, Taxus media; 10-DAB, 10-deacetylbaccatin III; DAB, baccatin III; M, Model group; TM-G, TM high dose group; TM-D, TM low dose group; TMQ-G, TMQ high dose group; TMQ-D, TMQ low dose group; T, Taxol group.
