# Supplementary material for: Effect of fermentation on the constituents in the branches and leaves of Taxus media and non-small cell lung cancer
Source: Front Pharmacol. 2024 Oct 23;15:1449498. doi: 10.3389/fphar.2024.1449498 (PMC11538029; doi:10.3389/fphar.2024.1449498)
Supplement: Supplementary file 1 [file DataSheet1.pdf]

Supplementary Material

1 Supplementary Figures and Tables

1.1 Supplementary Figures

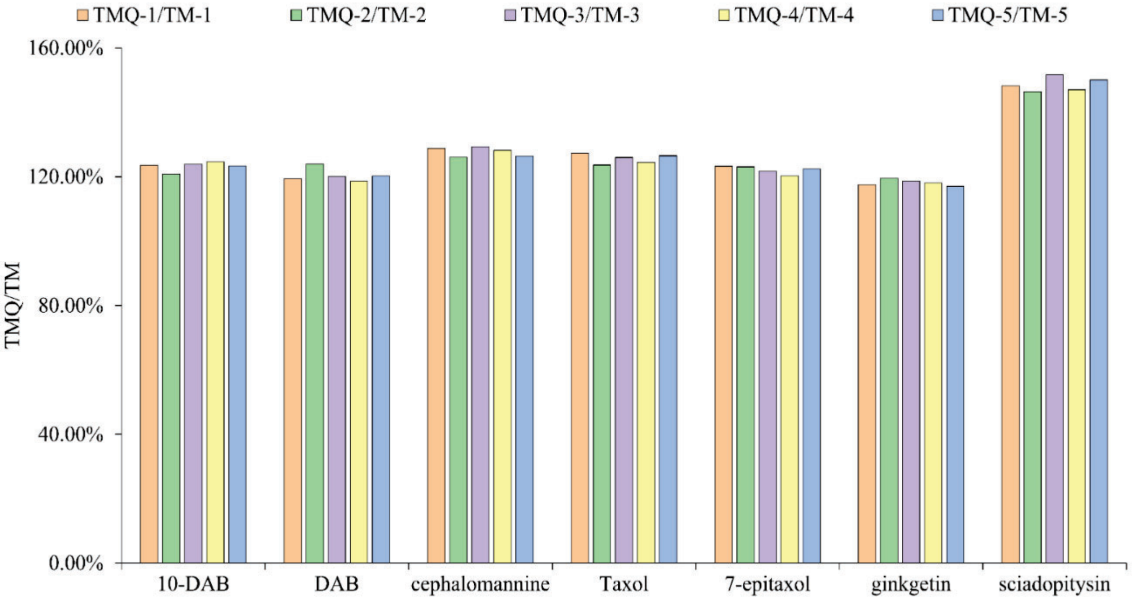

Supplementary Figure S1. The results of post-fermentation samples was higher than that pre-fermentation

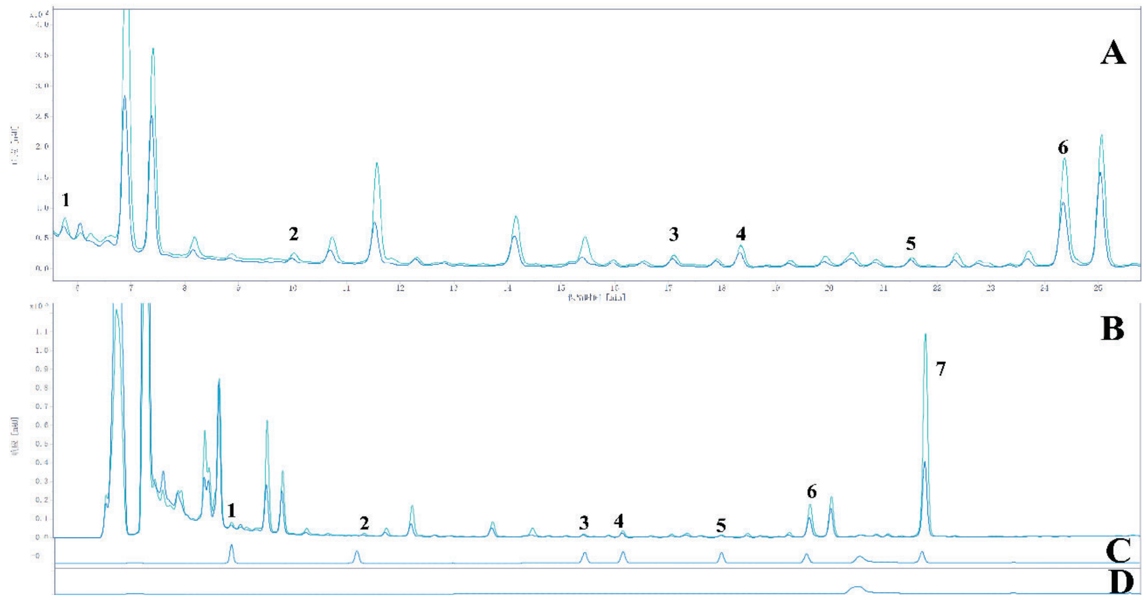

Supplementary Figure S2. HPLC profiles of TM and TMQ (A) Local amplified chromatograms of TM and TMQ samples from 5.5-25.5 min; (B) TM and TMQ samples superimposed on chromatograms (TMQ chromatograms in green, TM chromatograms in blue); C is“HB” mixed reference solution; D is“JC” blank solution; The numbers 1 through 7 are 10-DAB (1), DAB(2), cephalomannine(3), taxol(4), 7-epitaxol(5), ginkgetin(6), and sciadopitysin(7), respectively.

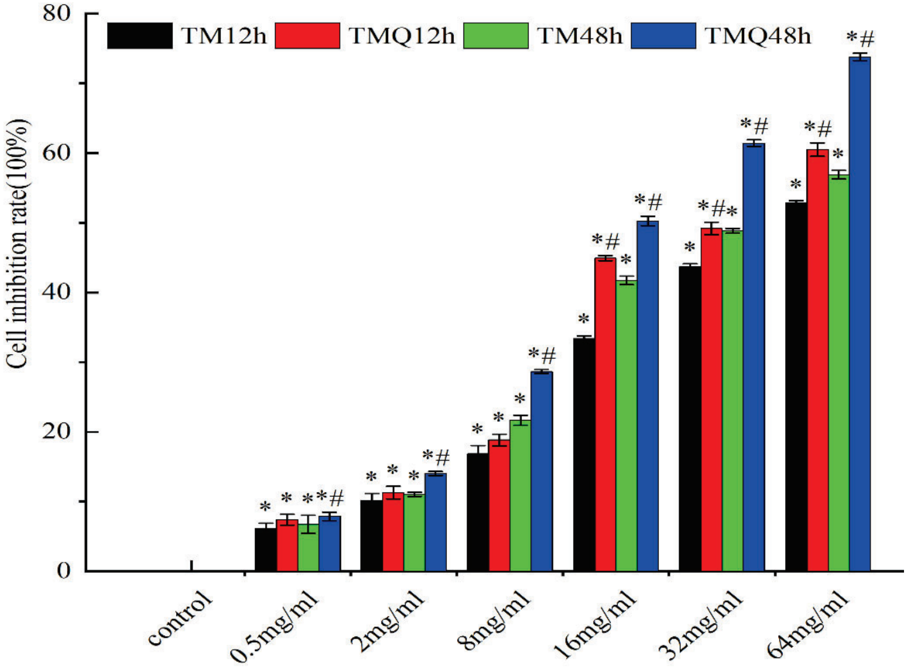

Supplementary Figure S3. Effect of methanol extracts from TM and TMQ on proliferation inhibition of A549 lung cancer cells. Data represent three replicates and are shown as mean ± standard deviation; * $p < 0.05$, compared with the control group; # $p < 0.05$, compared with the TM sample at the same action time and concentration.

1.2 Supplementary tables

Supplementary Table S1. Results of the linear survey of 10-DAB, DAB, cephalomannine, taxol, 7-epitaxol, ginkgetin and sciadopitysin.

Compound	Regression Equation	R2	Linear Range (μg/mL)
10-DAB	Y = 16,704.82 X + 12.37	0.9999	6.07~606.70
DAB	Y = 15,635.49 X + 3.69	0.9999	2.80~280.00
Cephalomannine	Y = 14,113.40 X + 0.49	0.9999	2.50~250.00
Taxol	Y = 14,957.48 X + 1.60	0.9999	1.63~163.30
7-epitaxol	Y = 15,766.64 X + 1.31	0.9999	0.67~66.70
Ginkgetin	Y = 29788.22 X + 15.23	0.9999	3.80~380.00
Sciadopitysin	Y = 36537.77 X + 40.12	0.9999	4.40~440.00
